# The Purinergic System and Glial Cells: Emerging Costars in Nociception

**DOI:** 10.1155/2014/495789

**Published:** 2014-09-03

**Authors:** Giulia Magni, Stefania Ceruti

**Affiliations:** ^1^Department of Pharmacological and Biomolecular Sciences, Università degli Studi di Milano, Via Balzaretti, 9-20133 Milan, Italy; ^2^Department of Drug Discovery and Development (D3), Italian Institute of Technology (IIT), Via Morego, 30-16163 Genoa, Italy

## Abstract

It is now well established that glial cells not only provide mechanical and trophic support to neurons but can directly contribute to neurotransmission, for example, by release and uptake of neurotransmitters and by secreting pro- and anti-inflammatory mediators. This has greatly changed our attitude towards acute and chronic disorders, paving the way for new therapeutic approaches targeting activated glial cells to indirectly modulate and/or restore neuronal functions. A deeper understanding of the molecular mechanisms and signaling pathways involved in neuron-to-glia and glia-to-glia communication that can be pharmacologically targeted is therefore a mandatory step toward the success of this new healing strategy. This holds true also in the field of pain transmission, where the key involvement of astrocytes and microglia in the central nervous system and satellite glial cells in peripheral ganglia has been clearly demonstrated, and literally hundreds of signaling molecules have been identified. Here, we shall focus on one emerging signaling system involved in the cross talk between neurons and glial cells, the purinergic system, consisting of extracellular nucleotides and nucleosides and their membrane receptors. Specifically, we shall summarize existing evidence of novel “druggable” glial purinergic targets, which could help in the development of innovative analgesic approaches to chronic pain states.

## 1. Chronic Pain: Neurons Need Costars to Be Sensitized

Several lines of evidence from basic pain research using diverse animal models indicate that an aberrant excitability of doreceptor agonists, such asrsal horn neurons evoked by peripheral sensory inputs is at the basis of both neuropathic and inflammatory pain [1, 2]. While neurons have long been considered the only cell type involved in pain development and transmission, recent studies have shown that pathologically altered neurotransmission requires communication with glial cells [3].

Microglia show a long-term response to a wide range of stimuli controlling physiological homeostasis, including peripheral nerve injury (PNI). In response to PNI, microglia activation in the spinal cord leads to cell hypertrophy, increase in cell number, and altered gene expression [4–6]. By responding to extracellular stimuli, activated glial cells evoke various cellular responses, such as production and release of bioactive factors including cytokines and neurotrophic factors [7], which in turn lead to the hyperexcitability of dorsal horn neurons and, consequently, to the development of neuropathic pain.

Not only spinal microglia but also astrocytes are involved in neuronal sensitization in the spinal cord. In fact, it has been hypothesized that microglia sense nerve damage or proalgogenic inputs from the periphery, and as a consequence they release several mediators that either act directly on dorsal horn neurons or promote reactive astrogliosis [8]. Reactive astrogliosis is a typical double-edged sword phenomenon, showing both neurotoxic and neuroprotective features, depending upon the type of injury and the basal conditions of the affected tissue [9]. This is possibly true also in pain models, where both astrocyte-derived proalgogenic and analgesic molecules have been detected [8].

Glial cells are directly involved in nociception not only in the central nervous system (CNS), but also in the periphery. In fact, a peculiar type of glial cells, named satellite glial cells (SGCs), is located in peripheral sensory ganglia, where they enwrap neuronal bodies, thus constituting distinct morphological and functional units [10]. In the past few years, their central role in the development and maintenance of chronic pain has been clearly demonstrated by several authors, reporting an increased expression and release of mediators such as interleukin-1*β* (IL-1*β*) and tumor necrosis factor alpha (TNF*α*) [11, 12], as well as their increased gap junction-mediated coupling following nerve injury [13]. In response to chronic pain, SGCs also upregulate the expression of glial fibrillary acidic protein (GFAP) and undergo cell division [14, 15].

The currently established role of glial cells in nociception suggests that the pharmacological manipulation of their reactivity might represent a groundbreaking analgesic strategy, as an alternative to a classical “neuron-centric” approach. It is therefore mandatory to identify the most promising key signaling pathways contributing to glial cell reaction to painful stimulation, which could be exploited pharmacologically. Here, we shall focus on the purinergic system, which in recent years has emerged as one of the most innovative and yet partially unexplored signaling systems controlling glial cell function under various pathological conditions, including pain. Far from being exhaustive in reviewing the exponentially growing number of publications in this field, this review aims at convincing the pain community that extracellular nucleotides and nucleosides are fundamental pro- and antinociceptive signals, mostly through their activity on peripheral and central glial cells.

## 2. Ionic Receptors Activated by Extracellular Nucleotides: The P2X Receptor Family

Extracellular nucleotides, mostly ATP but possibly also UTP, are costored in synaptic vesicles and actively released during physiological or pathological (i.e., excitotoxic) neurotransmission [16]. Glial sources of ATP have been also demonstrated, which contribute to physiological cell-to-cell communication [17]. Additionally, extracellular concentrations of nucleotides increase severalfold at sites of tissue damage, due to leakage of the intracellular nucleotide pool and degradation of nucleic acids [16, 17].

P2X receptors are membrane ion channels that open in response to the binding of extracellular ATP and elicit rapid responses (<10 ms), resulting in selective permeability to Na^+^, K^+^, and Ca^2+^ cations [18]. In vertebrates, seven genes encode P2X receptor subunits, which are 40–50% identical in their amino acidic sequence. Each subunit has two transmembrane domains, separated by an extracellular domain (~280 amino acids). Channels function as trimers of several subunits, and the extracellular domain is the portion containing the binding sites for nucleotides. To date, 7 homomeric (P2X1, P2X2, P2X3, P2X4, P2X5, P2X6, and P2X7), and 6 heteromeric P2X receptors (P2X1/2, P2X1/4, P2X1/5, P2X2/3, P2X2/6, and P2X4/6) have been identified, the latter showing specific pharmacological differences compared to their homomerical counterpart [18]. P2X receptors are differentially distributed among the cell types in different brain areas. Peripheral neurons mainly express the P2X3 and P2X2/3 receptor subtypes, which are involved in pain and temperature perception (see below) [19]. All P2X receptor subtypes can modulate membrane action potential. They are expressed either at the presynaptic level, regulating neurotransmitters release through the modulation of intracellular levels of Ca^2+^ ions, or postsynaptically, where they interact with ionotropic receptors (i.e., nicotinic; gamma-aminobutyric acid, GABA; N-Methyl-D-Aspartate, NMDA) via Ca^2+^-dependent kinases [16]. P2X receptors are involved in a variety of physiological processes, including the modulation of cardiac rhythm and contractility, vascular tone, platelet aggregation, macrophage activation, apoptosis, neuron-glia communication, and modulation of nociception [20].

## 3. P2X Receptors and Chronic Pain: Not Only a Matter of Neurons

Since the first publications demonstrating the involvement of ATP in pain transmission [21], the expression levels and functions of P2X receptors have been characterized in neurons from dorsal root ganglia (DRG) and in the trigeminal nerve [22]. All P2X receptor subtypes, with the exception of P2X7, are expressed in sensory neurons; among these, the P2X3 subtype showed the highest expression levels [23]. To date, the most important P2X receptor subtypes involved in pain transmission are (i) the P2X3 and P2X2/3 receptor subtypes, expressed by sensory neurons, (ii) the P2X4 receptor, expressed by CNS microglia, and (iii) the P2X7 receptor, expressed by CNS microglia and astrocytes and by SGCs in sensory ganglia [24].

## 4. Neuronal P2X3 Receptors: Key Players in Nociception

Although this review article is focused on glial purinergic receptors involved in pain transmission, a brief summary of data on neuronal P2X3 subtype, the most studied P2X receptor subtype involved in nociception, cannot be omitted. P2X3 receptors are selectively expressed at high levels in nociceptive primary sensory neurons in trigeminal, nodose, and dorsal root ganglia [25]. The selective expression of P2X3 receptors in these areas involved in pain transmission suggested that they may be crucial in processing pain signals, and several studies have evaluated the relationships between P2X3 receptors and pain sensation.

P2X3 receptors have been initially cloned and functionally characterized from rat DRG [26], showing their unique expression in small and medium size sensory neurons and their absence in large diameter neurons [27, 28]. On the other hand, P2X3 has never been found to be expressed by glial cells, by either immunohistochemical analysis or calcium imaging [29]. P2X3 immunoreactivity has also been observed in other structures indirectly involved in nociceptive transmission, such as the nucleus of the solitary tract, and the spinal trigeminal nucleus [30].

A number of* in vitro* and* in vivo* studies have evaluated the relationships between P2X3 receptors and pain transmission. Local injection of P2X3 receptor agonists, such as ATP or* α*,*β*-methyleneATP, into the hind paws of rats induced nociceptive behaviors and reduced thermal and mechanical thresholds [31, 32]. During inflammation and chronic constriction injuries, ATP concentrations and neuronal expression of P2X3 receptors are upregulated in DRG, trigeminal ganglion (TG), and spinal cord [33, 34]. In animal models of inflammatory and neuropathic pain, P2X3 receptor knock-out or its downregulation by antisense oligonucleotides or by short interfering RNA significantly attenuated painful behaviors, including tactile allodynia and mechanical hyperalgesia [35]. In addition, the nonselective P2X3 receptor antagonists TNP-ATP [2′,3′-O-(2,4,6 trinitrophenyl) adenosine-5′-triphosphate] and PPADS pyridoxal phosphate-6-azophenyl-2′,4′ disulfonic acid reduced the excitability of DRG neurons and neuropathic pain [36]. Overall, these findings suggest that neuronal P2X3 receptors play a crucial role in the development and maintenance of chronic pain.

Despite the above-mentioned results, more recent studies have shown a transient decrease in P2X3 expression in a model of partial damage of the mental nerve (belonging to the mandibular branch of the trigeminal nerve), or no significant expression changes following resection of the lingual nerve [37]. It is possible that these differences reflect the type of lesion: after a constriction (i.e., application of a ligature) an increase in the expression levels is observed, while nerve injury or full axotomy leads to reduction or no changes of receptor expression. More importantly, it has been observed that variations in P2X3 expression levels are modulated at different time points after the lesion; a transient decrease in the expression was observed in the first two weeks after nerve injury, followed by an increase during the following weeks, paralleled by the increased expression of glial cell-derived neurotrophic factor (GDNF) and the generation of new neuronal branches (sprouting) in the damaged sensory areas [38]. Therefore, the timing and the type of lesion are critical for the observed results. Finally, it is also worth mentioning that P2X3 receptors are involved in the mechanism of action of known analgesics, as recently shown for the nonsteroidal anti-inflammatory drug naproxen, which inhibits P2X3 activation in the TG [39]. Moreover, it is now emerging that bioactive molecules contained in analgesic preparation from traditional Chinese medicine (such as tetramethylpyrazine, sodium ferulate, and puerarin) can inhibit P2X3-mediated transmission in primary afferent neurons [40].

## 5. Glial P2X4 and P2X7 Receptors

Microglia-neuron communication is bidirectional, and several lines of evidence implicate ATP as a critical signaling molecule [41–43]. Microglia expression of P2X receptors is restricted to the P2X4 and P2X7 subtypes [42, 44], whose activation evokes current responses, increases intracellular [Ca^2+^], and causes the release of signaling molecules affecting neuronal functions [45, 46].

The central role of P2X4 receptor subtype in neuropathic pain was first reported by [47]. In this paper, authors showed that intrathecal injection of TNP-ATP, acting as antagonist at P2X1-4 receptor subtypes, reverted tactile allodynia in nerve-injured rats. On the contrary, treatment with PPADS, antagonist at P2X1-3, 5, and 7 but not at P2X4 receptor, had no effect. Authors therefore concluded that the main P2X receptor subtype involved in central responses to peripheral nerve injury is the P2X4 receptor, and the development and maintenance of tactile allodynia require its activation. To confirm this hypothesis, targeting P2X4 receptors with antisense oligonucleotides [47] or the genetic deletion of the* P2rx4* gene [48] significantly attenuated nerve injury-induced pain phenotypes. Moreover, the development of tactile allodynia correlated with a progressive increase in spinal P2X4 receptor expression, which is generally low in naïve CNS [48].

Further immunohistochemical analysis of wild-type (wt) mice, and studies on transgenic mice selectively expressing the green fluorescent protein in microglial cells revealed that this cell population rather than neurons or astrocytes was expressing the P2X4 receptor subtype [48] and that the increase in P2X4 receptor expression temporally correlated with the development of tactile allodynia. P2X4 receptor upregulation was a consequence, rather than a cause of microglia activation; in fact, preventing the increase in P2X4 receptor did not affect the expression of protein markers of microglia activation, such as complement receptor 3 or the ionized calcium binding adaptor molecule-1 (Iba-1) [47, 48]. Interestingly, activation of microglial P2X4 receptor is sufficient to elicit pain, since the intrathecal injection of P2X4 receptor-stimulated cultured microglia induced mechanical allodynia in naïve animals [41, 47, 49]. Taken together, pharmacological, genetic, and behavioral findings indicate that activation of P2X4 receptor subtype expressed by spinal microglia is both necessary and sufficient to induce tactile allodynia after peripheral nerve injury.

**Figure 1:**
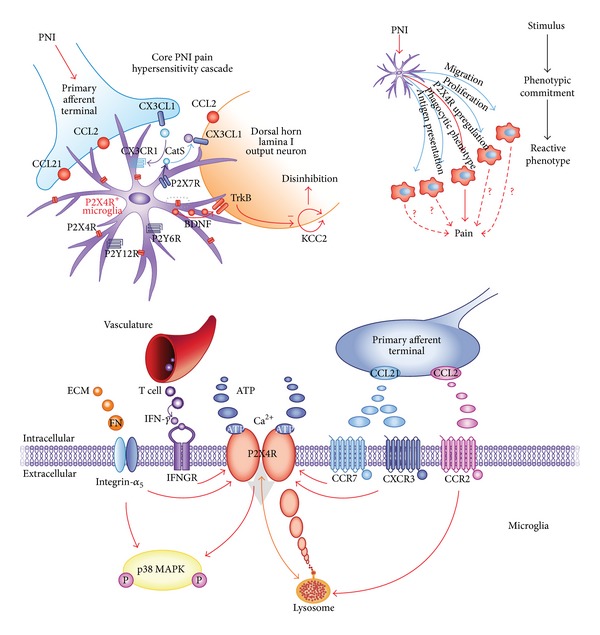
Central role of microglial P2X4 receptors in mediating a molecular cascade leading to hypersensitivity after peripheral nerve injury (PNI). The upper left cartoon shows the neuron-to-microglia cross talk triggered by neuronal injury, leading to the appearance of an “activated” microglia phenotype, which expresses higher levels of the P2X4 receptor. The dotted rectangle is expanded in the lower panel, showing the complex molecular network responsible for P2X4 receptor upregulation and involving chemokines, growth factors, and integrins. Activation of the P2X4 receptor ultimately leads to the recruitment and activation of p38 MAP kinase. Reproduced from [56] with permission from Nature Publishing Group.

Concerning the bidirectional cross talk between activated microglia and dorsal horn neurons, the chemokine (C-C motif) ligand 21 (CCL21) released from injured neurons has been demonstrated to act as an upstream activator of P2X4 receptor [50], in close collaboration with interferon *γ*, a cytokine converting resting spinal microglia into its activated state [51], and tryptase, a protease released from mast cells that activates proteinase-activated receptor 2 in microglia [52] (Figure 1). Another critical element for the upregulation of P2X4 receptors expression is the extracellular matrix molecule fibronectin, which modulates the transcriptional and posttranscriptional levels of P2X4 receptor expression in microglia through the activity of Lyn kinase and the downstream activation of intracellular signaling pathways involving phosphatidylinositol 3-kinase- (PI3 K-) Akt and mitogen-activated protein kinase kinase- (MAPK kinase, MEK-) extracellular signal-regulated kinase (ERK) [49, 53, 54]. The implications of this wide modulation network and whether these elements are connected into a common pathway controlling P2X4 receptor expression are still not known.

On the other way around, it has been reported that the activation of microglial P2X4 receptor promoted the synthesis and release of brain-derived neurotrophic factor (BDNF), through the activation of p38 MAPkinase [48, 55]. BDNF in turn altered the transmembrane anion gradient in a subpopulation of dorsal horn lamina I neurons, through the downregulation of the neuronal chloride transporter KCC2. This makes GABA and glycine act as depolarizing rather than hyperpolarizing neurotransmitters, causing an aberrant nociceptive output that contributes to the development of neuropathic pain [41, 56].

In addition to the P2X4 subtype, microglial cells also express functional P2X7 receptors. The P2X7 receptor is a unique member of the P2X receptor family, since it is only activated by high concentrations of ATP (>100 *μ*M) and can open a much larger membrane pore than any other P2X channel [57]. Stimulation of P2X7 receptors is implicated in microglia response to inflammation, release of proinflammatory cytokines, like IL-1*β*, and proliferation [58], underlining the involvement of this receptor subtype in inflammatory diseases and pain.

Although P2X7 receptors are expressed on a wide variety of cell types, evidence for involvement of microglial P2X7 receptors in chronic neuropathic pain stems from findings that its activation causes the p38 MAPkinase-dependent release of IL-1*β* and cathepsin S, which contribute to the maintenance of mechanical hypersensitivity in the spinal cord [59, 60]. Additional hints on the role of P2X7 receptors in neuropathic pain have been provided by the demonstration of reduced pain sensitivity in P2X7 receptor-deficient mice [61] and by the analgesic effects exerted by P2X7 receptor antagonists [62, 63]. In fact, authors demonstrated the absence of inflammatory and neuropathic hypersensitivity In fact, in mice lacking the P2X7 receptor, authors demonstrated the absence of inflammatory and neuropathic hypersensitivity after both mechanical and thermal stimuli, accompanied by a reduction of adjuvant-induced increases in IL-1*β*, IL-6, IL-10, and macrophage chemoattractant protein-1, while normal nociceptive processing is preserved [61]. Moreover, P2X7 receptor was upregulated in human DRG and injured nerves from chronic neuropathic pain patients [61]. Recent studies also showed that P2X7 receptor expression is increased in the ipsilateral spinal cord after sciatic nerve ligation and is highly restricted to microglia; moreover, chronic constriction injury- (CCI-) induced mechanical allodynia and thermal hypersensitivity are reversed by intrathecal administration of the selective P2X7 antagonist Brilliant Blue G [64].

Interestingly, a correlation between single nucleotide polymorphisms (SNPs) within the coding sequence of the* P2rx7 *gene and the sensitivity to chronic pain has been provided in both mice and humans. In fact, mice in which P2X7 receptors have impaired pore formation due to the P451L mutation showed less allodynia than mice with the wt pore-forming* P2rx7 *allele. Indeed, administration of a peptide, which blocked pore formation but not cation channel activity, selectively reduced nerve injury and inflammatory allodynia only in wt mice. Moreover, in two independent human chronic pain cohorts, a cohort with pain after mastectomy and a cohort with osteoarthritis, authors observed a genetic association between lower pain intensity and the hypofunctional His270 allele of P2X7 receptor, further suggesting that selectively targeting P2X7 pore formation may be a new strategy for individualizing the treatment of chronic pain [65].

Several lines of evidence suggest a functional convergence between the signaling pathways activated by P2X4 and P2X7 receptors in microglia. As previously mentioned, p38 MAPkinase is involved in both the P2X4 receptor-mediated release of BDNF and the P2X7-induced release of IL-1*β* and cathepsin S (see above). Moreover, a recent paper showed that phosphorylation of p38 MAPK via P2X7 receptor may induce tactile allodynia/hyperalgesia in an orofacial pain model based on the CCI of the infraorbital nerve (CCI-ION), which is most likely mediated by soluble TNF-*α* released by microglia [66]. Thus, activation of p38 MAPK may be a critical mechanistic step of convergence in the P2X7 and P2X4 receptor signaling pathways in neuropathic pain. P2X7 and P2X4 receptors also interact to form complexes with unique channel properties [44, 67]. This raises the interesting possibility that structural and functional interactions, as well as converging signaling pathways, might be critical cellular mechanisms that underlie the involvement of microglial P2X7 and P2X4 receptors in neuropathic pain. The expression of P2X4 and P2X7 receptors subtype in a variety of cell types involved in pain transmission represents a promising target for pharmacological intervention. For example, locally administered oxidized ATP, an irreversible inhibitor of P2X7 receptors, had an antihyperalgesic effect on complete Freund's adjuvant-induced mechanical hyperalgesia (paw pressure test) [68].

An important role has been also disclosed for the P2X7 receptor subtype in the modulation of reactive astrogliosis. Following trauma, an upregulation of its expression on astrocytes has been demonstrated, and its activation by extracellular ATP or by synthetic agonists led to the release of proinflammatory IL-1*β*, ATP itself, and glutamate [17]. These molecules can further sustain and propagate astrocytic reaction to injury and can also sensitize nearby neurons in the spinal cord, thus contributing to nociception. More recently, the P2X7-mediated astrocytic release of dynorphins has been demonstrated in the spinal cord [69], thus further corroborating the notion of a direct involvement of astrocytic P2X7 receptor subtype in nociception.

The role of glial P2X7 in nociception is not restricted to the CNS, since recent data established its expression in SGCs, the specialized population of glial cells that enwrap neuronal bodies in peripheral sensory ganglia [10]. In sensory ganglia, ATP released from neuronal somata engages P2X7 receptors expressed by surrounding SGCs [12], which in turn inhibits back neuronal algogenic P2X3 receptors [70]. Additional P2X receptor subtypes are upregulated in SGCs upon exposure to proinflammatory conditions [71], but their role in nociception still remains elusive.

## 6. G Protein-Coupled Receptors Activated by Extracellular Nucleotides: The P2Y Receptor Family

The first P2Y receptor (currently named as the P2Y_1_ subtype) was cloned in 1993 [76, 77], and since then a total of eight human P2Y receptor subtypes have been identified: the P2Y_1_, P2Y_2_, P2Y_4_, P2Y_6_, P2Y_11_, P2Y_12_, P2Y_13_, and P2Y_14_ receptors [78]. The various P2Y receptor subtypes are functionally coupled to different G proteins, and, consequently, downstream signaling pathways, either phospholipase C, with inositol 3 phosphate (IP_3_) formation and intracellular calcium increase, or adenylyl cyclase, leading to modulation of cyclic adenosine monophosphate (cAMP) production [78]. From a pharmacological point of view, P2Y receptors can be broadly subdivided into four groups based on their responsivity to nucleotides: (i) adenine nucleotide-preferring receptors, mainly responding to ADP and ATP; this group includes human and rodent P2Y_1_, P2Y_12_, and P2Y_13_, and human P2Y_11_; (ii) uracil nucleotide-preferring receptors, including the human P2Y_4_ and P2Y_6_ responding to either UTP or UDP; (iii) receptors of mixed selectivity (the human and rodent P2Y_2_, the rodent P2Y_4_ and, possibly, P2Y_11_); and (iv) the P2Y_14_ receptor, responding to both UDP and sugar nucleotides (mainly UDP-glucose and UDP-galactose) [78, 79].

## 7. Glial P2Y Receptors and Chronic Pain: Still Elusive but Promising Targets for New Analgesic Strategies

Unlike the key and quite well defined role played by P2X receptors in pain transmission, controversial information is currently available concerning P2Y receptor contribution to nociception. Nevertheless, it is well known that these receptors are widely expressed by neurons and glial cells in both the central and the peripheral nervous systems [16], thus leading to hypothesize that their activity is integrated in the complex molecular network associated with the transmission of nociceptive signals. The comprehension of their role in pain pathogenesis is therefore of paramount importance for the discovery of new potential drug targets for pain therapy.

As for P2Y receptors expressed by sensory neurons, the P2Y_1_ receptor subtype is often coexpressed with the P2X3 receptor, acting as its functional antagonist in DRG [80], while the P2Y_12,13_ subtypes can inhibit N-type voltage-gated calcium channels [81]. Taken together, these data suggest an overall analgesic profile for ADP-sensitive P2Y receptor activation. Surprisingly, P2Y_1_ receptors were also reported to mediate hyperalgesia, possibly through the sensitization of transient receptor potential vanilloid 1 (TRPV_1_) channels [82], thus suggesting that the functional outcome of P2Y_1_ receptor activation could depend upon the specific tissue conditions. The same hyperalgesic potentiation of TRPV1 receptors has been later demonstrated for the UTP-sensitive P2Y_2_ receptor subtype in rat lumbar DRGs [83].

Increasing evidence is highlighting a role for glial P2Y receptors in chronic pain. Microglia express a wide range of P2Y receptors (P2Y_1,2,4,6,12_), with the P2Y_6_ and P2Y_12_ receptor subtypes mediating chemotaxis and migration towards the site of damage [43, 84]. To date, only the P2Y_12_ receptor subtype is clearly known to be involved in neuropathic pain onset. In fact, in response to peripheral nerve injury, microglial P2Y_12_ receptor expression was upregulated, and its activation was critical for microglial engulfment of myelinated axons in the spinal dorsal horn [43]. Moreover, pharmacological blockade, antisense knockdown of P2Y_12_ receptor expression, or genetic deletion of the* P2ry12* gene suppresses both mechanical allodynia and thermal hyperalgesia in nerve-injured rats [85, 86]. Conversely, intrathecal administration of the P2Y_12_ receptor agonist 2Me-SADP elicits pain behavior in naïve rats, mimicking what is observed in nerve-injured rats [85]. Moreover, mRNA not only for the P2Y_12_, but also for the P2Y_6,13,14_ receptor subtypes was upregulated in microglia in the ipsilateral spinal cord following nerve injury, and a mixture of P2Y_6_, P2Y_12_, and P2Y_13_ selective antagonists showed a longer suppressive effect on pain behavior compared to every single treatment itself [87].

The P2Y_6_ receptor can be considered a sensor for microglial cell phagocytosis by sensing diffusible UDP signals in primary cultured microglial cells [84]. Although microglial P2Y_6_ receptors expression markedly increases in the spinal cord after peripheral nerve injury [87], its specific role under neuropathic pain conditions still remains unclear. As already mentioned, ATP-gated P2X4 receptor channels expressed in spinal microglia actively participate in central sensitization, making their functional regulation a key process in chronic pain pathologies (see above); interestingly, it has been recently reported that P2X4 receptors function is inhibited by the P2Y_6_ receptor subtype, highlighting its possible critical role in regulating neuropathic pain-induced microglial responses [88].

Although not directly associated with pain signaling to date, it is conceivable that also P2Y receptors expressed by astrocytes could significantly contribute to nociception, especially in the spinal cord. P2Y receptors have been directly implicated in the modulation of reactive astrogliosis since the 1990s, also thanks to the contribution of our laboratory (for reviews, see [9, 89]). In particular, activation of the P2Y_1_ receptor subtype has been directly correlated with the release of prostaglandins and other proinflammatory mediators [90, 91] and directly or indirectly correlated with the transmission of painful sensations. Targeting astrocytic P2Y receptors could therefore represent an innovative approach to pain, specifically neuropathic pain, through the modulation of astroglial inputs controlling second order spinal neurons firing.

Very few data are currently available on the role of the P2Y receptor family in SGCs, and the vast majority of the available data concerns expression rather than functional studies (for reviews see [75, 92]). Under basal conditions, SGCs in the trigeminal ganglion express functional P2Y_1,2,4,6,13_ receptor subtypes [28, 93], whereas glial P2Y expression in DRG cells has been only evaluated at the mRNA level [94], with the exception of P2Y_1_ receptors protein which has been detected by immunofluorescence studies [70].

Within the TG, the close interplay between the purinergic system and other classical pain transducing signals has been recently elucidated, starting from the observation that chronic exposure of TG cultures to the algogenic mediator bradykinin induced the upregulation of P2Y-mediated calcium signaling in SGCs [28, 95], thus prompting us to speculate that P2Y receptors expressed by SGCs could represent possible pharmacological targets for the development of innovative therapies against migraine and trigeminal pain in general (for review see [75]). From then on,* in vitro* and* in vivo* studies have revealed that those specific glial P2Y receptors potentiated under proalgogenic conditions are the P2Y_1_ and P2Y_2_ subtypes (Magni et al., manuscript in preparation). These results have been partially confirmed by a recent paper showing that P2Y_2_ receptor inhibition induces analgesia in a rat model of neuropathic pain based on CCI-ION [96], although authors mostly focused on P2Y_2_ receptors expressed by trigeminal sensory neurons. Altogether, these data highlight the key role of P2Y purinergic receptors, and especially of the P2Y_2_ subtype, as promising targets for the development of new analgesic drugs.

**Figure 2:**
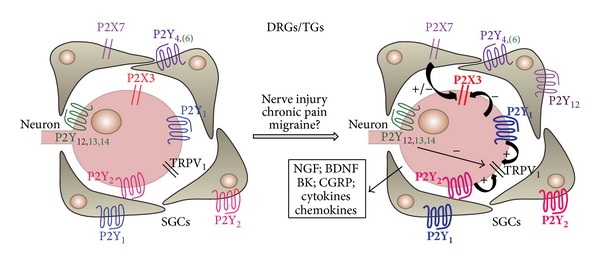
Plasticity of purinergic signaling within sensory ganglia upon proinflammatory conditions. The cartoon schematically summarizes currently available data on the modulation of P2 receptor expression by proalgogenic and proinflammatory conditions in sensory ganglia. An increased line weight indicates receptor upregulation, which has been observed in both sensory neurons and surrounding satellite glial cells. Some receptor subtypes (i.e., glial P2Y_12_ receptor subtype) are only detected upon algogenic conditions. Activation of P2 receptors by extracellular nucleotides leads to the modulation of other systems involved in nociception, like TRPV1 receptors, and in the release of additional cytokines, growth factors, and other substances (see box), further contributing to the development and maintenance of a proalgogenic milieu. Reproduced from [75] with permission from Elsevier.

An emerging role for the glial P2Y_12_ receptor subtype has been demonstrated in an animal model of trigeminal neuropathic pain [97]. In fact, although not expressed in the TG upon control conditions [14], expression of the P2Y_12_ receptor subtype was detected in activated SGCs after unilateral lingual nerve crush. Interestingly, administration of a P2Y_12_-selective antagonist significantly reverted glial cell activation and alleviated animals' sensitivity to mechanical and heat stimulation of the tongue [97]. These data further sustain the hypothesis that a shift and/or upregulation in purinergic receptor expression can be observed within sensory ganglia, and especially on SGCs, upon proalgogenic conditions (Figure 2). This observation opens up the possibility that drugs selectively targeting specific purinergic receptors could exert their analgesic activity at the site of receptor upregulation, thus limiting the occurrence of systemic and unwanted side effects.

## 8. Adenosine Exerts Analgesic Effects through Its Glial Receptors: An Additional Component of Its Neuromodulatory Actions

Adenosine is the major metabolite of the ectonucleotidase-mediated sequential dephosphorylation of ATP. Therefore, it cannot be classified as a true neurotransmitter, but rather as a neuromodulator, through the activation of 4 subtypes of G protein-coupled receptors, that is, the A_1_, A_2A_, A_2B_, and A_3_ adenosine receptor subtypes, collectively referred to as P1 receptors [98]. The temporal relationship between ATP release and subsequent adenosine generation from its hydrolysis guarantees the generation of a feedback inhibitory loop, which overall counteracts the excitatory effects of ATP. This is particularly true for the A_1_ receptor subtype, which is coupled with inhibition of cAMP synthesis and with the activation of potassium conductance, with consequent inhibition of neuronal firing and neurotransmitter release, reduction of heart rate, and other inhibitory effects.

A key role for the A_1_ receptor subtype in the modulation of painful sensations has been hypothesized more than 15 years ago. Both spinal and peripheral A_1_ receptors have been found directly involved in the analgesic effects exerted by several known drugs, like tramadol, acetaminophen, and amitriptyline, often in close interconnection with 5-HT_7_ serotonin receptors [99–101]. Moreover, recent studies also suggest that the analgesic efficacy of some herbal preparations long utilized in traditional Chinese medicine can be at least in part due to a modulation of the adenosine system. In fact, norisoboldine (the major active alkaloid component isolated from the dry roots of* Lindera aggregata* often utilized for chest and abdomen pain, indigestion, cold hernia, and frequent urination) was shown to promote analgesia in both the formalin and the writhing tests in rodents through the activation of A_1_ receptor subtype [102].

The cell population expressing the A_1_ receptor subtype involved in pain transmission was not clearly identified, until the publication of papers suggesting its microglial identity. An A_1_ receptor subtype selective agonist was in fact shown to alleviate neuropathic pain and to reduce spinal microglia activation in the spared nerve injury rat model [103]. Most interestingly, authors later demonstrated that exposure to proalgogenic ATP induced a significant increase in A_1_ receptor subtype expression in microglia, whose activation in turn led to inhibition of microglial phenotypic changes and to the reduction of the ability of microglia to promote neuronal firing [104]. Thus, it can be envisaged that microglial A_1_ receptors are upregulated and engaged to counteract the algogenic actions induced by massive ATP release at injury or inflammation sites.

The role of additional adenosine receptor subtypes in nociception is much less clear. Conflicting results have been obtained by administering either A_2A_ receptor subtype-selective agonists or antagonists in animal pain models, showing both analgesia and hyperalgesia [105]. In fact, it has been demonstrated that A_2A_ receptor knock-out mice have a reduced sensitivity to pronociceptive stimuli, which seems to be mediated by the loss of pronociceptive A_2A_ receptors on DRG primary sensory neurons [106]. It should be mentioned, however, that microglia express A_2A_ receptors, which are directly involved in the modulation of the release of proalgogenic stimuli, like nerve growth factor (NGF), prostaglandin E2 (PGE_2_), and nitric oxide (NO) [107]. Based on the above-mentioned data, it can therefore be envisaged that selective A_2A_ receptor antagonists could counteract the proinflammatory and proalgogenic features of microglial cells and ultimately prove to be useful as antialgogenic agents, at least in inflammatory pain conditions. Conversely, the intrathecal injection of a selective A_2A_ receptor agonist produced a significant and long-lasting reversal of mechanical allodynia and thermal hyperalgesia consequent to the CCI of the sciatic nerve [108]. A reduction of glial cell activation and a significant upregulation in the production of anti-inflammatory IL-10 in the spinal cord were also observed, thus suggesting that activating rather than inhibiting the A_2A_ receptor subtype could represent a better option for neuropathic pain states [108].

Finally, the availability of selective agonist ligands has allowed demonstrating an antialgogenic role for the A_3_ receptor subtype in neuropathic pain induced either by the CCI of the sciatic nerve or by chemotherapic agents in rodents [109]. The cellular localization of the target receptors involved in nociception has not been directly identified, although it has been demonstrated that the A_3_ receptor subtype is involved in ADP-mediated microglia chemotaxis and process extension [110].

Although their direct role in pain transmission has not been proven yet, it is worth mentioning that all the four adenosine receptor subtypes are expressed by spinal cord and brain astrocytes and significantly contribute to the modulation of reactive astrogliosis (for review see [111]). It is therefore conceivable that they can participate to astrocytic modulation of neuronal functions, especially under chronic pain conditions, when high amounts of adenosine are produced as a consequence of the hydrolysis of extracellular nucleotides and of the leakage of nucleic acids from damaged cells [111]. To date the role of adenosine receptors in peripheral ganglia has not been studied in detail.

A major limitation to the systemic use of drugs acting on adenosine receptors is represented by their wide distribution, especially in the CNS and in the cardiovascular system, which could account for significant and harmful side effects. Specifically, activation of the A_1_ adenosine receptors in the CNS could lead to inhibition of locomotor activity and catalepsy, whereas in the heart severe bradycardia and atrioventricular block may occur [112]. Acting on the A_2A_ subtype with selective agonists may lead to a significant drop in blood pressure, accompanied by tachycardia [112]. Conversely, despite the significant increase of circulating histamine that was observed in mice, clinical studies in cancer patients have shown no significant side effects after administration of selective A_3_ receptor agonists [112].

## 9. When Will an Analgesic Drug Targeting Glial Purinergic Receptors Be Finally Available?

The incomplete and still growing list of scientific studies reviewed in this paper clearly demonstrates that targeting glial purinergic receptors might significantly improve the currently available armamentarium of analgesic drugs to be utilized in chronic pain state, still lacking a satisfactory pharmacological control. Nevertheless, to date no new purinergic drug entities have reached the market. This is mostly due to (i) the widespread expression of all the different receptor subtypes, which renders the discrimination between beneficial and side effects extremely complicated, (ii) the lack of good subtype-selective agents, and (iii) when selective ligands are available, the lack of an acceptable route of administration to humans.

Concerning the most studied purinergic receptor in nociception, the neuronal P2X3 subtype, no clinical studies were available so far, despite the massive preclinical* in vivo* and* in vitro* efforts. This was mainly due to the lack of selective antagonists with a chemical structure suitable for administration to humans [37]. Very recently, however, two clinical trials employing the new orally active P2X3-selective antagonist AF-219 [113] were concluded (see Table 1), although results have not been disclosed yet. Recently, the extensive use of high-throughput screening followed by a hit-to-lead program has prompted a number of pharmaceutical companies to identify highly selective, potent, and metabolically stable small-molecule inhibitors for both P2X4 and P2X7 receptor, which provide a novel therapeutic approach to the treatment of pain and inflammatory disorders [114, 115]. Nevertheless, no significant results have emerged from the first clinical trials performed with different P2X7-selective antagonists (Table 1), thus suggesting that the role of this receptor subtype should be reconsidered, at least in rheumatoid arthritis-related pain.

As for P2Y receptors, although important progress in exploring structure-activity relationships has been recently achieved with the publication of the first crystal structure of a member of the family [116], most of the P2Y receptor subtypes are still lacking potent and selective synthetic agonists and antagonists [78], which has greatly hampered both research in this field, and the translation of* in vitro* data to* in vivo* and clinical settings. Additionally, the only P2Y-selective antagonists available so far, acting on the P2Y_12_ receptor subtype, are widely utilized for their strong antiplatelet activity [116], which poses serious concerns about possible side effects in case of their administration for painful conditions.

Finally, in the case of the A_1_ adenosine receptor subtype, its widespread distribution has greatly limited the preclinical and clinical use of the available A_1_-selective agonists, due to significant systemic side effects (see above). Recently, it has been demonstrated that a novel and potent A_1_ receptor allosteric modulator, named TRR469, exhibits antinociceptive activity in the formalin and writhing tests in mice, without showing the usual side effects of A_1_-selective agonists (see above) [117].

It is also worth reaffirming that expression of purinergic receptor on glial cells is highly plastic and can therefore significantly change during the course of chronic pain conditions (Figure 2). For example, it has been hypothesized that the purinergic system is fundamental for the so-called “modal shift” of microglia, namely, their acquisition of different phenotypes over time, based on the time-dependent modification of the expression profile of P1 and P2 receptors [118]. This additional fascinating, but complicating, issue should be taken into consideration when deciding which is the best target option for the development of an antihyperalgesic rather than an antiallodynic purinergic-based agent.

Therefore, based on these promises and pitfalls, a closer collaboration among pharmacology, biochemistry, molecular biology, and pharmaceutical chemistry is highly recommended to clearly identify and target the most promising “druggable” receptors, which could be successfully utilized in preclinical and clinical studies to accelerate the process towards new therapeutic options for chronic pain patients.

## Figures and Tables

**Table 1:** Summary of past and current clinical trials evaluating purinergic ligands in pain.

Name of ligand	Company	Clinical trial number	Type of pain	Main outcomes
AF219 (P2X3 antagonist)	Afferent Pharmaceuticals	NCT01554579	Osteoarthritis of the knee	No published results yet
		NCT01569438	Interstitial cystitis/bladder pain syndrome	No published results yet

CE-224,535 (P2X7 antagonist)	Pfizer	NCT00628095	Rheumatoid arthritis	CE-224,535 was not efficacious, compared with placebo, for the treatment of RA in patients with an inadequate response to metotrexate [72]
		NCT00418782	Osteoarthritis of the knee	CE-224,535 failed to demonstrate efficacy in a 2-week study of knee pain in osteoarthritis patients

AZD9056	AstraZeneca	NCT00520572	Rheumatoid arthritis	AZD9056 does not have significant efficacy in the treatment of RA [73]

GSK1482160 (P2X7 antagonist)	GlaxoSmithKline	NCT00849134	“First-in-human” study to assess the pharmacokinetic profile of the drug	No major safety or tolerability concerns were identified in this study. Nevertheless, monitoring the release of IL-1*β* in subjects led to the decision to discontinue drug development for inflammatory pain conditions [74]

Adenosine (e.v.)	Wake Forest School of Medicine	NCT00349921	Neuropathic pain (comparison with clonidine)	No published results yet
	Xsira Pharmaceuticals	NCT00298636	Perioperative pain (hysterectomy/myomectomy)	No published results yet

GW493838	GlaxoSmithKline	NCT00376454	Neuropathic pain as a result of postherpetic neuralgia or nerve injury	No published results yet

## References

[1] Woolf CJ, Salter MW (2000). Neuronal plasticity: increasing the gain in pain. *Science*.

[2] Xu F, Li Y, Li S (2014). Complete Freund's adjuvant-induced acute inflammatory pain could be attenuated by triptolide via inhibiting spinal glia activation in rats. *Journal of Surgical Research*.

[3] Ji R, Berta T, Nedergaard M (2013). Glia and pain: is chronic pain a gliopathy?. *Pain*.

[4] Tsuda M, Inoue K, Salter MW (2005). Neuropathic pain and spinal microglia: a big problem from molecules in “small” glia. *Trends in Neurosciences*.

[5] Suter MR, Wen Y, Decosterd I, Ji R (2007). Do glial cells control pain?. *Neuron Glia Biology*.

[6] Kazuhide I, Makoto T (2009). Microglia and neuropathic pain. *Glia*.

[7] Inoue K (2006). ATP receptors of microglia involved in pain. *Novartis Foundation Symposium*.

[8] Tiwari V, Guan Y, Raja SN (2014). Modulating the delicate glial-neuronal interactions in neuropathic pain: promises and potential caveats. *Neuroscience & Biobehavioral Reviews*.

[9] Buffo A, Rolando C, Ceruti S (2010). Astrocytes in the damaged brain: molecular and cellular insights into their reactive response and healing potential. *Biochemical Pharmacology*.

[10] Hanani M (2010). Satellite glial cells in sympathetic and parasympathetic ganglia: in search of function. *Brain Research Reviews*.

[11] Takeda M, Tanimoto T, Kadoi J (2007). Enhanced excitability of nociceptive trigeminal ganglion neurons by satellite glial cytokine following peripheral inflammation. *Pain*.

[12] Zhang X, Chen Y, Wang C, Huang L-YM (2007). Neuronal somatic ATP release triggers neuron-satellite glial cell communication in dorsal root ganglia. *Proceedings of the National Academy of Sciences of the United States of America*.

[13] Ledda M, Blum E, de Palo S, Hanani M (2009). Augmentation in gap junction-mediated cell coupling in dorsal root ganglia following sciatic nerve neuritis in the mouse. *Neuroscience*.

[14] Villa G, Ceruti S, Zanardelli M (2010). Temporomandibular joint inflammation activates glial and immune cells in both the trigeminal ganglia and in the spinal trigeminal nucleus. *Molecular Pain*.

[15] Donegan M, Kernisant M, Cua C (2013). Satellite glial cell proliferation in the trigeminal ganglia after chronic constriction injury of the infraorbital nerve. *Glia*.

[16] Abbracchio MP, Burnstock G, Verkhratsky A, Zimmermann H (2009). Purinergic signalling in the nervous system: an overview. *Trends in Neurosciences*.

[17] Burnstock G, Krügel U, Abbracchio MP, Illes P (2011). Purinergic signalling: from normal behaviour to pathological brain function. *Progress in Neurobiology*.

[18] North RA, Jarvis MF (2013). P2X receptors as drug targets. *Molecular Pharmacology*.

[19] Khmyz V, Maximyuk O, Teslenko V, Verkhratsky A, Krishtal O (2008). P2X_3_ receptor gating near normal body temperature. *Pflugers Archiv European Journal of Physiology*.

[20] Alexander SPH, Benson HE, Faccenda E (2013). The concise guide to PHARMACOLOGY 2013/14: ligand-gated ion channels. *British Journal of Pharmacology*.

[21] Bleehen T, Keele CA (1977). Observations on the algogenic actions of adenosine compounds on the human blister base preparation. *Pain*.

[22] Dunn PM, Zhong Y, Burnstock G (2001). P2X receptors in peripheral neurons. *Progress in Neurobiology*.

[23] Kim YS, Paik SK, Cho YS (2008). Expression of P2X3 receptor in the trigeminal sensory nuclei of the rat. *Journal of Comparative Neurology*.

[24] Burnstock G (2009). Purines and sensory nerves. *Handbook of Experimental Pharmacology*.

[25] Cho JH, Jung KY, Jung Y (2013). Design and synthesis of potent and selective P2X3 receptor antagonists derived from PPADS as potential pain modulators. *European Journal of Medicinal Chemistry*.

[26] Chen CC, Akopian AN, Sivilotti L, Colquhoun D, Burnstock G, Wood JN (1995). A P2X purinoceptor expressed by a subset of sensory neurons. *Nature*.

[27] North RA (2004). P2X3 receptors and peripheral pain mechanisms. *The Journal of Physiology*.

[28] Ceruti S, Fumagalli M, Villa G, Verderio C, Abbracchio MP (2008). Purinoceptor-mediated calcium signaling in primary neuron-glia trigeminal cultures. *Cell Calcium*.

[29] Hullugundi SK, Ferrari MD, van den Maagdenberg AMJM, Nistri A (2013). The mechanism of functional up-regulation of P2X3 receptors of trigeminal sensory neurons in a genetic mouse model of familial hemiplegic migraine type 1 (FHM-1). *PLoS ONE*.

[30] Llewellyn-Smith IJ, Burnstock G (1998). Ultrastructural localization of P2X3 receptors in rat sensory neurons. *NeuroReport*.

[31] Tsuda M, Koizumi S, Kita A, Shigemoto Y, Ueno S, Inoue K (2000). Mechanical allodynia caused by intraplantar injection of P2X receptor agonist in rats: involvement of heteromeric P2X2/3 receptor signaling in capsaicin-insensitive primary afferent neurons. *Journal of Neuroscience*.

[32] Chen Y, Li GW, Wang C, Gu Y, Huang LM (2005). Mechanisms underlying enhanced P2X receptor-mediated responses in the neuropathic pain state. *Pain*.

[33] Kage K, Niforatos W, Zhu CZ, Lynch KJ, Honore P, Jarvis MF (2002). Alteration of dorsal root ganglion P2X3 receptor expression and function following spinal nerve ligation in the rat. *Experimental Brain Research*.

[34] Cheng RD, Tu WZ, Wang WS (2013). Effect of electroacupuncture on the pathomorphology of the sciatic nerve and the sensitization of P2X_3_ receptors in the dorsal root ganglion in rats with chronic constrictive injury. *Chinese Journal of Integrative Medicine*.

[35] Barclay J, Patel S, Dorn G (2002). Functional downregulation of P2X3 receptor subunit in rat sensory neurons reveals a significant role in chronic neuropathic and inflammatory pain. *The Journal of Neuroscience*.

[36] Honore P, Mikusa J, Bianchi B (2002). TNP-ATP, a potent P2X3 receptor antagonist, blocks acetic acid-induced abdominal constriction in mice: comparison with reference analgesics. *Pain*.

[37] Ford AP (2012). In pursuit of P2X3 antagonists: novel therapeutics for chronic pain and afferent sensitization. *Purinergic Signalling*.

[38] Taylor AMW, Ribeiro-da-Silva A (2011). GDNF levels in the lower lip skin in a rat model of trigeminal neuropathic pain: implications for nonpeptidergic fiber reinnervation and parasympathetic sprouting. *Pain*.

[39] Hautaniemi T, Petrenko N, Skorinkin A, Giniatullin R (2012). The inhibitory action of the antimigraine nonsteroidal anti-inflammatory drug naproxen on P2X3 receptor-mediated responses in rat trigeminal neurons. *Neuroscience*.

[40] Liang S, Xu C, Li G, Gao Y (2010). P2X receptors and modulation of pain transmission: focus on effects of drugs and compounds used in traditional Chinese medicine. *Neurochemistry International*.

[41] Coull JAM, Beggs S, Boudreau D (2005). BDNF from microglia causes the shift in neuronal anion gradient underlying neuropathic pain. *Nature*.

[42] Jarvis MF (2010). The neural-glial purinergic receptor ensemble in chronic pain states. *Trends in Neurosciences*.

[43] Maeda M, Tsuda M, Tozaki-Saitoh H, Inoue K, Kiyama H (2010). Nerve injury-activated microglia engulf myelinated axons in a P2Y_12_ signaling-dependent manner in the dorsal horn. *Gila*.

[44] Trang T, Beggs S, Salter MW (2012). ATP receptors gate microglia signaling in neuropathic pain. *Experimental Neurology*.

[45] Trang T, Beggs S, Salter MW (2012). Brain-derived neurotrophic factor from microglia: a molecular substrate for neuropathic pain. *Neuron Glia Biology*.

[46] Bianco MR, Cirillo G, Petrosino V (2012). Neuropathic pain and reactive gliosis are reversed by dialdehydic compound in neuropathic pain rat models. *Neuroscience Letters*.

[47] Tsuda M, Shigemoto-Mogami Y, Koizumi S (2003). P2X4 receptors induced in spinal microglia gate tactile allodynia after nerve injury. *Nature*.

[48] Ulmann L, Hatcher JP, Hughes JP (2008). Up-regulation of P2X4 receptors in spinal microglia after peripheral nerve injury mediates BDNF release and neuropathic pain. *Journal of Neuroscience*.

[49] Tsuda M, Toyomitsu E, Komatsu T (2008). Fibronectin/integrin system is involved in P2X(4) receptor upregulation in the spinal cord and neuropathic pain after nerve injury. *Glia*.

[50] Biber K, Tsuda M, Tozaki-Saitoh H (2011). Neuronal CCL21 up-regulates microglia P2X4 expression and initiates neuropathic pain development. *EMBO Journal*.

[51] Tsuda M, Masuda T, Kitano J, Shimoyama H, Tozaki-Saitoh H, Inoue K (2009). IFN-*γ* receptor signaling mediates spinal microglia activation driving neuropathic pain. *Proceedings of the National Academy of Sciences of the United States of America*.

[52] Yuan H, Zhu X, Zhou S (2010). Role of mast cell activation in inducing microglial cells to release neurotrophin. *Journal of Neuroscience Research*.

[53] Nasu-Tada K, Koizumi S, Tsuda M, Kunifusa E, Inoue K (2006). Possible involvement of increase in spinal fibronectin following peripheral nerve injury in upregulation of microglial P2X4, a key molecule for mechanical allodynia. *Glia*.

[54] Tsuda M, Tozaki-Saitoh H, Masuda T (2008). Lyn tyrosine kinase is required for P2X4 receptor upregulation and neuropathic pain after peripheral nerve injury. *GLIA*.

[55] Trang T, Beggs S, Wan X, Salter MW (2009). P2X4-receptor-mediated synthesis and release of brain-derived neurotrophic factor in microglia is dependent on calcium and p38-mitogen-activated protein kinase activation. *Journal of Neuroscience*.

[56] Beggs S, Trang T, Salter MW (2012). P2X4R + microglia drive neuropathic pain. *Nature Neuroscience*.

[57] North RA (2002). Molecular physiology of P2X receptors. *Physiological Reviews*.

[58] Monif M, Burnstock G, Williams DA (2010). Microglia: proliferation and activation driven by the P2X7 receptor. *International Journal of Biochemistry and Cell Biology*.

[59] Clark AK, Staniland AA, Marchand F, Kaan TKY, McMahon SB, Malcangio M (2010). P2X7-dependent release of interleukin-1*β* and nociception in the spinal cord following lipopolysaccharide. *Journal of Neuroscience*.

[60] Clark AK, Wodarski R, Guida F, Sasso O, Malcangio M (2010). Cathepsin S release from primary cultured microglia is regulated by the P2X7 receptor. *Glia*.

[61] Chessell IP, Hatcher JP, Bountra C (2005). Disruption of the P2X7 purinoceptor gene abolishes chronic inflammatory and neuropathic pain. *Pain*.

[62] McGaraughty S, Chu KL, Namovic MT (2007). P2X7-related modulation of pathological nociception in rats. *Neuroscience*.

[63] Perez-Medrano A, Donnelly-Roberts DL, Honore P (2009). Discovery and biological evaluation of novel cyanoguanidine P2X7 antagonists with analgesic activity in a rat model of neuropathic pain. *Journal of Medicinal Chemistry*.

[64] He W, Cui J, Du L (2012). Spinal P2X 7 receptor mediates microglia activation-induced neuropathic pain in the sciatic nerve injury rat model. *Behavioural Brain Research*.

[65] Sorge RE, Trang T, Dorfman R (2012). Genetically determined P2X7 receptor pore formation regulates variability in chronic pain sensitivity. *Nature Medicine*.

[66] Ito G, Suekawa Y, Watanabe M (2013). P2X7 receptor in the trigeminal sensory nuclear complex contributes to tactile allodynia/hyperalgesia following trigeminal nerve injury. *European Journal of Pain*.

[67] Guo C, Masin M, Qureshi OS, Murrell-Lagnado RD (2007). Evidence for functional P2X4/P2X7 heteromeric receptors. *Molecular Pharmacology*.

[68] Dell’Antonio G, Quattrini A, Dal Cin E, Fulgenzi A, Ferrero ME (2002). Antinociceptive effect of a new P2Z/P2X7 antagonist, oxidized ATP, in arthritic rats. *Neuroscience Letters*.

[69] Wahlert A, Funkelstein L, Fitzsimmons B, Yaksh T, Hook V (2013). Spinal astrocytes produce and secrete dynorphin neuropeptides. *Neuropeptides*.

[70] Chen Y, Zhang X, Wang C, Li G, Gu Y, Huang LM (2008). Activation of P2X_7_ receptors in glial satellite cells reduces pain through downregulation of P2X_3_ receptors in nociceptive neurons. *Proceedings of the National Academy of Sciences of the United States of America*.

[71] Kushnir R, Cherkas PS, Hanani M (2011). Peripheral inflammation upregulates P2X receptor expression in satellite glial cells of mouse trigeminal ganglia: a calcium imaging study. *Neuropharmacology*.

[72] Stock TC, Bloom BJ, Wei N (2012). Efficacy and safety of CE-224,535, an antagonist of P2X_7_ receptor, in treatment of patients with rheumatoid arthritis inadequately controlled by methotrexate. *Journal of Rheumatology*.

[73] Keystone EC, Wang MM, Layton M, Hollis S, McInnes IB (2012). Clinical evaluation of the efficacy of the P2X7 purinergic receptor antagonist AZD9056 on the signs and symptoms of rheumatoid arthritis in patients with active disease despite treatment with methotrexate or sulphasalazine. *Annals of the Rheumatic Diseases*.

[74] Ali Z, Laurijssens B, Ostenfeld T (2013). Pharmacokinetic and pharmacodynamic profiling of a P2X_7_ receptor allosteric modulator GSK1482160 in healthy human subjects. *British Journal of Clinical Pharmacology*.

[75] Magni G, Ceruti S (2013). P2Y purinergic receptors: new targets for analgesic and antimigraine drugs. *Biochemical Pharmacology*.

[76] Lustig KD, Shiau AK, Brake AJ, Julius D (1993). Expression cloning of an ATP receptor from mouse neuroblastoma cells. *Proceedings of the National Academy of Sciences of the United States of America*.

[77] Webb TE, Simon J, Krishek BJ (1993). Cloning and functional expression of a brain G-protein-coupled ATP receptor. *FEBS Letters*.

[78] Abbracchio MP, Burnstock G, Boeynaems J (2006). International Union of Pharmacology LVIII: update on the P2Y G protein-coupled nucleotide receptors: from molecular mechanisms and pathophysiology to therapy. *Pharmacological Reviews*.

[79] Carter RL, Fricks IP, Barrett MO (2009). Quantification of Gi-mediated inhibition of adenylyl cyclase activity reveals that UDP is a potent agonist of the human P2Y_14_ receptor. *Molecular Pharmacology*.

[80] Gerevich Z, Müller C, Illes P (2005). Metabotropic P2Y_1_ receptors inhibit P2X3 receptor-channels in rat dorsal root ganglion neurons. *European Journal of Pharmacology*.

[81] Malin SA, Molliver DC (2010). Gi- and Gq-coupled ADP (P2Y) receptors act in opposition to modulate nociceptive signaling and inflammatory pain behavior. *Molecular Pain*.

[82] Tominaga M, Wada M, Masu M (2001). Potentiation of capsaicin receptor activity by metabotropic ATP receptors as a possible mechanism for ATP-evoked pain and hyperalgesia. *Proceedings of the National Academy of Sciences of the United States of America*.

[83] Moriyama T, Iida T, Kobayashi K (2003). Possible involvement of P2Y_2_ metabotropic receptors in ATP-induced transient receptor potential vanilloid receptor 1-mediated thermal hypersensitivity. *Journal of Neuroscience*.

[84] Koizumi S, Shigemoto-Mogami Y, Nasu-Tada K (2007). UDP acting at P2Y_6_ receptors is a mediator of microglial phagocytosis. *Nature*.

[85] Kobayashi K, Yamanaka H, Fukuoka T, Dai Y, Obata K, Noguchi K (2008). P2Y_12_ receptor upregulation in activated microglia is a gateway of p38 signaling and neuropathic pain. *Journal of Neuroscience*.

[86] Tozaki-Saitoh H, Tsuda M, Miyata H, Ueda K, Kohsaka S, Inoue K (2008). P2Y_12_ receptors in spinal microglia are required for neuropathic pain after peripheral nerve injury. *Journal of Neuroscience*.

[87] Kobayashi K, Yamanaka H, Yanamoto F, Okubo M, Noguchi K (2012). Multiple P2Y subtypes in spinal microglia are involved in neuropathic pain after peripheral nerve injury. *Glia*.

[88] Bernier LP, Ase AR, Boué-Grabot É, Séguéla P (2013). Inhibition of P2X4 function by P2Y6 UDP receptors in microglia. *Glia*.

[89] Di Virgilio F, Ceruti S, Bramanti P, Abbracchio MP (2009). Purinergic signalling in inflammation of the central nervous system. *Trends in Neurosciences*.

[90] Xia M, Zhu Y (2011). Signaling pathways of ATP-induced PGE_2_ release in spinal cord astrocytes are EGFR transactivation-dependent. *GLIA*.

[91] Boccazzi M, Rolando C, Abbracchio MP (2014). Purines regulate adult brain subventricular zone cell functions: contribution of reactive astrocytes. *Glia*.

[92] Kobayashi K, Yamanaka H, Noguchi K (2013). Expression of ATP receptors in the rat dorsal root ganglion and spinal cord. *Anatomical Science International*.

[93] Villa G, Fumagalli M, Verderio C, Abbracchio MP, Ceruti S (2010). Expression and contribution of satellite glial cells purinoceptors to pain transmission in sensory ganglia: an update. *Neuron Glia Biology*.

[94] Kobayashi K, Fukuoka T, Iyamanaka H (2006). Neurons and glial cells differentially express P2Y receptor mRNAs in the rat dorsal root ganglion and spinal cord. *Journal of Comparative Neurology*.

[95] Ceruti S, Villa G, Fumagalli M (2011). Calcitonin gene-related peptide-mediated enhancement of purinergic neuron/glia communication by the algogenic factor bradykinin in mouse trigeminal ganglia from wild-type and R192Q Ca_v_2.1 knock-in mice: Implications for basic mechanisms of migraine pain. *Journal of Neuroscience*.

[96] Li N, Lu ZY, Yu LH (2014). Inhibition of G protein-coupled P2Y_2_ receptor induced analgesia in a rat model of trigeminal neuropathic pain. *Molecular Pain*.

[97] Katagiri A, Shinoda M, Honda K, Toyofuku A, Sessle BJ, Iwata K (2012). Satellite glial cell P2Y_12_ receptor in the trigeminal ganglion is involved in lingual neuropathic pain mechanisms in rats. *Molecular Pain*.

[98] Chen JF, Eltzschig HK, Fredholm BB (2013). Adenosine receptors as drug targets-what are the challenges?. *Nature Reviews Drug Discovery*.

[99] Sawynok J, Reid AR, Liu J (2013). Spinal and peripheral adenosine A_1_ receptors contribute to antinociception by tramadol in the formalin test in mice. *European Journal of Pharmacology*.

[100] Liu J, Reid AR, Sawynok J (2013). Antinociception by systemically-administered acetaminophen (paracetamol) involves spinal serotonin 5-HT7 and adenosine A_1_ receptors, as well as peripheral adenosine A1 receptors. *Neuroscience Letters*.

[101] Liu J, Reid AR, Sawynok J (2013). Spinal serotonin 5-HT7 and adenosine A_1_ receptors, as well as peripheral adenosine A_1_ receptors, are involved in antinociception by systemically administered amitriptyline. *European Journal of Pharmacology*.

[102] Gao X, Lu Q, Chou G (2014). Norisoboldine attenuates inflammatory pain via the adenosine A_1_ receptor. *European Journal of Pain*.

[103] Luongo L, Petrelli R, Gatta L (2012). 5'-Chloro-5'-deoxy-(±)-ENBA, a potent and selective adenosine A_1_ receptor agonist, alleviates neuropathic pain in mice through functional glial and microglial changes without affecting motor or cardiovascular functions. *Molecules*.

[104] Luongo L, Guida F, Imperatore R (2014). The A_1_ adenosine receptor as a new player in microglia physiology. *Glia*.

[105] Ferré S, Diamond I, Goldberg SR (2007). Adenosine A_2A_ receptors in ventral striatum, hypothalamus and nociceptive circuitry: implications for drug addiction, sleep and pain. *Progress in Neurobiology*.

[106] Li L, Hao JX, Fredholm BB, Schulte G, Wiesenfeld-Hallin Z, Xu XJ (2010). Peripheral adenosine A_2A_ receptors are involved in carrageenan-induced mechanical hyperalgesia in mice. *Neuroscience*.

[107] Pocock JM, Kettenmann H (2007). Neurotransmitter receptors on microglia. *Trends in Neurosciences*.

[108] Loram LC, Taylor FR, Strand KA (2013). Intrathecal injection of adenosine 2A receptor agonists reversed neuropathic allodynia through protein kinase (PK)A/PKC signaling. *Brain, Behavior, and Immunity*.

[109] Chen Z, Janes K, Chen C (2012). Controlling murine and rat chronic pain through A_3_ adenosine receptor activation. *The FASEB Journal*.

[110] Ohsawa K, Sanagi T, Nakamura Y, Suzuki E, Inoue K, Kohsaka S (2012). Adenosine A_3_ receptor is involved in ADP-induced microglial process extension and migration. *Journal of Neurochemistry*.

[111] Daré E, Schulte G, Karovic O, Hammarberg C, Fredholm BB (2007). Modulation of glial cell functions by adenosine receptors. *Physiology and Behavior*.

[112] Antonioli L, Csóka B, Fornai M (2014). Adenosine and inflammation: what's new on the horizon?. *Drug Discovery Today*.

[113] Ford AP (2012). P2X3 antagonists: novel therapeutics for afferent sensitization and chronic pain. *Pain Management*.

[114] Furber M, Alcaraz L, Bent JE (2007). Discovery of potent and selective adamantane-based small-molecule P2X7 receptor antagonists/interleukin-1*β* inhibitors. *Journal of Medicinal Chemistry*.

[115] Hernandez-Olmos V, Abdelrahman A, El-Tayeb A, Freudendahl D, Weinhausen S, Müller CE (2012). N-substituted phenoxazine and acridone derivatives: structure-activity relationships of potent P2X4 receptor antagonists. *Journal of Medicinal Chemistry*.

[116] Zhang K, Zhang J, Gao ZG (2014). Structure of the human P2Y_12_ receptor in complex with an antithrombotic drug. *Nature*.

[117] Vincenzi F, Targa M, Romagnoli R (2014). TRR469, a potent A_1_ adenosine receptor allosteric modulator, exhibits anti-nociceptive properties in acute and neuropathic pain models in mice. *Neuropharmacology*.

[118] Koizumi S, Ohsawa K, Inoue K, Kohsaka S (2013). Purinergic receptors in microglia: functional modal shifts of microglia mediated by P2 and P1 receptors. *Glia*.

